# Microbiomes as the new keystone for life sciences development

**DOI:** 10.1111/1751-7915.13424

**Published:** 2019-05-07

**Authors:** Zulema Udaondo, Carlos Molina‐Santiago

**Affiliations:** ^1^ Department of Biomedical Informatics University of Arkansas for Medical Sciences Little Rock AR 72205 USA; ^2^ Departamento de Microbiología Instituto de Hortofruticultura Subtropical y Mediterránea ‘La Mayora’ Universidad de Málaga ‐ Consejo Superior de Investigaciones Científicas (IHSM‐UMA‐CSIC) Universidad de Málaga Bulevar Louis Pasteur 31 (Campus Universitario de Teatinos) 29071 Málaga Spain

It has not been long since we abandoned the dogma asserting that all internal organs are sterile. In fact, all eukaryotic organisms are considered now as holobionts representing complex collaborations between the entire microbiome of each eukaryote and its innate cells. These dwellers are mainly found in specific ‘hot spots’ habitats that contain larger microbe concentrations such as the gut, mouth, skin, vagina and lung airways. All of these body sites possess fairly stable bacterial communities with their own distinctive taxonomic composition (Gilbert *et al*., 2018). Recent advances in the state of the art of DNA sequencing, the development of ‐omic technologies and the improvement of data analysis tools have enabled the microbiome research to become one of the most dynamic areas of research of our time (Schmidt *et al*., 2018) even changing our concept of eukaryotic organisms as ‘superorganisms’ (Segre and Salafsky, 2016; Kutschera, 2018).

Although there is a widespread agreement about the important role that our microbial symbionts play in many aspects of our health and their link in many complicated syndromes as obesity (Turnbaugh and Gordon, 2009), Crohn's disease (Eckburg and Relman, 2007), Parkinson's disease, autism, depression (Knight *et al*., 2017), several metabolic disorders (Spencer *et al*., 2011) and even in our behaviour (Johnson and Foster, 2018), there is still a lack of knowledge on the definition of ‘normality’ or stable state of the microbial composition in each of its ecosystems (Costello *et al*., 2009). This is due to important features of the microbiomes in their colonization niches, as their taxonomic profiles and the relative abundances of each of the species, which differ across individuals and can even change over time (Califf *et al*., 2014).

Besides humans, microbiome studies are also becoming popular in other species and different ecological niches, such as non‐human mammals, fishes and marine environments. The methodology employed in all these studies, however, remains largely the same regardless of the host. Interesting examples of non‐human microbial population studies are the works recently published in Microbial Biotechnology highlighting the implication of the imbalance of microbial flora from different anatomic sites with lesions and diseases (Huang *et al*., 2018), or the relatedness between changes in diet with changes in the microbiome in different species (Simons *et al*., 2018; Walburn *et al*., 2019). As stated above, linking changes in microbial communities with human diseases is a hot spot in microbial population studies. In this sense, the work done by Huang and colleagues (Huang *et al*., 2018) shows the importance of the lung microbial flora in mammals, and more specifically its relationship with lung's chronic lesions in swines. In this paper, authors firstly establish the bacterial communities found in healthy swine lungs, mainly dominated by *Proteobacteria* (47.3%), *Firmicutes* (23.8%) and *Bacteroidetes* (18.7%) phyla. Comparison with injured swine's lungs suggests a significant reduction of the microbial diversity and the implication of enriched bacterial species found in severe‐lesion lungs, such as *Mycoplasma hyopneaumoniae* and *Mycoplasma flocculare*, in the development of lesions in this organ (Huang *et al*., 2018). As authors assert in this work, reduction of the microbial diversity has been associated with other human diseases such as cystic fibrosis, chronic obstructive pulmonary disease, Crohn's disease and irritable bowel syndrome.

In addition to the remarkable advances emphasizing the importance of microbiomes in human and animal diseases, microbiomes are also attracting interest from economical and biotechnological points of view. Modulation of human and animal microbiomes by food and dietary supplements has been commonly observed in our society for many years claiming to ‘promote intestinal health’. Furthermore, efforts have been done in the aquaculture industry in an attempt to better understand economically valuable fish species and to improve fish growth and development.

Examples of that are the investigations done by Simons *et al*. (2018) and Walburn *et al*. (2019) demonstrating the effect of diet in the microbial gut composition of oysters and measuring the changes in the microbial community of the intestine of Yellowtail Kingfish (*Seriola lalandi*) at different stages of development respectively. The work by Simons *et al*. (2018) addresses important questions such as the impact of the living microorganisms, present in the algal culture used as feedstock for feeding the Pacific oyster (*Crassostrea gigas)*, in shaping the digestive bacterial community of the oyster, or in other words, what is more influential, prebiotics or probiotics? In this study, authors show that the dominant taxa in the oyster's faecal microbiome are distinct from the corresponding algal microbiomes used to feed the oyster, suggesting that the nutritional profile of the algal culture used as feedstock plays a more important role on the gut microbiome composition than the associated bacterial communities present in the algal culture. However, as expected, changes in diet show differences in oyster's faecal microbial populations, suggesting a direct relationship with the algal feed used in each diet. The study also suggests a role of the external environment in shaping the internal bacterial community, being likewise significantly different from that present on the algal feed used (Simons *et al*., 2018).

However, studies performed by Walburn *et al*. (2019) focused in Yellowtail Kingfish gut microbiome, highlight the importance of microbes from diet as well as those present in the rearing water, during the first stages of the fish larvae development and growth, showing a direct involvement of both the feed‐associated and environmental bacteria in the gut microbiome. Moreover, this study let to identify a core microbial community, mainly composed of *Streptococcus*,* Enterococcus* and *Lactobacillus* species, representing the 56% of the whole community, which seems to be stable at all developmental stages and, as stated in other studies, is related with healthy fish gut microbiome. On the other hand, stage‐specific communities identified are related with changes in diet, from live feeds during the larvae stage to formulated pellets, thus suggesting that the composition of the gut microbiome of aquaculture animals may be influenced as well by the introduction of formulated feedstock, regardless of their age. Further analyses of gene function associated with the gut microbiome at the different stages of the fish larval development show changes in the levels of representation of different metabolic pathways which seems to be also related to changes in the nutritional profile associated to the different diets. An interesting note is, for example, the high representation of secondary metabolite pathways during the early larval stage of the Yellowtail Kingfish that would suggest a role of gut‐associated microbes on the production of antibiotics that could protect the host against pathogenic invaders.

The past decades can be considered as the golden era for microbiome research, and great progress has been done so far. The application of microbial population analyses has demonstrated promising prospects with therapeutic, agriculture and aquaculture applications to improve health and increase productivity in agriculture. Furthermore, new technologies and developments in the area of machine learning are being developed to provide a deeper insight into the chemistry of microbiomes and its link with dysbiosis diseases. Although it is not clear yet the direction of this correlation, there is no doubt nowadays that the microbiome research and its applications will change the way we live and will serve to improve our lives.

## Conflict of interests

None declared.

## References

[mbt213424-bib-0001] Califf, K. , Gonzalez, A. , Knight, R. , and Caporaso, J.G. (2014) The human microbiome: getting personal. Microbe 9: 410–415.

[mbt213424-bib-0002] Costello, E.K. , Lauber, C.L. , Hamady, M. , Fierer, N. , Gordon, J.I. , and Knight, R. (2009) Bacterial community variation in human body habitats across space and time. Science 326: 1694–1697. 19892944 10.1126/science.1177486 PMC3602444

[mbt213424-bib-0003] Eckburg, P.B. , and Relman, D.A. (2007) The role of microbes in crohn's disease. Clin Infect Dis 44: 256–262. 17173227 10.1086/510385

[mbt213424-bib-0004] Gilbert, J.A. , Blaser, M.J. , Caporaso, J.G. , Jansson, J.K. , Lynch, S.V. , and Knight, R. (2018) Current understanding of the human microbiome. Nat Med 24: 392–400. 29634682 10.1038/nm.4517 PMC7043356

[mbt213424-bib-0005] Huang, T. , Zhang, M. , Tong, X. , Chen, J. , Yan, G. , Fang, S. , *et al* (2018) Microbial communities in swine lungs and their association with lung lesions. Microb Biotechnol 12: 289–304. 10.1111/1751-7915.13353 30556308 PMC6389860

[mbt213424-bib-0006] Johnson, K.V.‐A. , and Foster, K.R. (2018) Why does the microbiome affect behaviour? Nat Rev Microbiol 16: 647. 29691482 10.1038/s41579-018-0014-3

[mbt213424-bib-0007] Knight, R. , Callewaert, C. , Marotz, C. , Hyde, E.R. , Debelius, J.W. , McDonald, D. , and Sogin, M.L. (2017) The microbiome and human biology. Annu Rev Genomics Hum Genet 18: 65–86. 28375652 10.1146/annurev-genom-083115-022438

[mbt213424-bib-0008] Kutschera, U. (2018) Systems biology of eukaryotic superorganisms and the holobiont concept. Theory Biosci 137: 117–131. 29948922 10.1007/s12064-018-0265-6

[mbt213424-bib-0009] Schmidt, T.S.B. , Raes, J. , and Bork, P. (2018) The human gut microbiome: from association to modulation. Cell 172: 1198–1215. 29522742 10.1016/j.cell.2018.02.044

[mbt213424-bib-0010] Segre, J.A. , and Salafsky, N. (2016) Hominid superorganisms. Science 353: 350–351. 27463659 10.1126/science.aag2788

[mbt213424-bib-0011] Simons, A.L. , Churches, N. , and Nuzhdin, S. (2018) High turnover of faecal microbiome from algal feedstock experimental manipulations in the Pacific oyster (*Crassostrea gigas*). Microb Biotechnol 11: 848–858. 29749083 10.1111/1751-7915.13277 PMC6116748

[mbt213424-bib-0012] Spencer, M.D. , Hamp, T.J. , Reid, R.W. , Fischer, L.M. , Zeisel, S.H. , and Fodor, A.A. (2011) Association between composition of the human gastrointestinal microbiome and development of fatty liver with choline deficiency. Gastroenterology 140: 976–986. 21129376 10.1053/j.gastro.2010.11.049 PMC3049827

[mbt213424-bib-0013] Turnbaugh, P.J. , and Gordon, J.I. (2009) The core gut microbiome, energy balance and obesity. J Physiol 587: 4153–4158. 19491241 10.1113/jphysiol.2009.174136 PMC2754355

[mbt213424-bib-0014] Walburn, W.J. , Wemheuer, B. , Thomas, T. , Copeland, E. , O'Connor, W. , Booth, M. , *et al* (2019) Diet and diet‐associated bacteria shape early microbiome development in Yellowtail Kingfish (*Seriola lalandi*). Microb Biotechnol 12: 275–288. 30506824 10.1111/1751-7915.13323 PMC6389859

